# New Empirical Bayes Models to Jointly Analyze Multiple RNA-Sequencing Data in a Hypophosphatasia Disease Study

**DOI:** 10.3390/genes15040407

**Published:** 2024-03-26

**Authors:** Dawson Kinsman, Jian Hu, Zhi Zhang, Gengxin Li

**Affiliations:** 1 Department of Mathematics and Statistics, University of Michigan-Dearborn, Dearborn, MI 48128, USA; dkinsman@umich.edu; 2 Manufacturing Systems Engineering, University of Michigan-Dearborn, Dearborn, MI 48128, USA; jianhu@umich.edu; 3 Department of Natural Sciences, University of Michigan-Dearborn, Dearborn, MI 48128, USA; zhizhan@umich.edu

**Keywords:** hypophosphatasia, RNA-sequencing, empirical Bayes, feature selection

## Abstract

Hypophosphatasia is a rare inherited metabolic disorder caused by the deficiency of tissue-nonspecific alkaline phosphatase. More severe and early onset cases present symptoms of muscle weakness, diminished motor coordination, and epileptic seizures. These neurological manifestations are poorly characterized. Thus, it is urgent to discover novel differentially expressed genes for investigating the genetic mechanisms underlying the neurological manifestations of hypophosphatasia. RNA-sequencing data offer a high-resolution and highly accurate transcript profile. In this study, we apply an empirical Bayes model to RNA-sequencing data acquired from the spinal cord and neocortex tissues of a mouse model, individually, to more accurately estimate the genetic effects without bias. More importantly, we further develop two integration methods, weighted gene approach and weighted *Z* method, to incorporate two RNA-sequencing data into a model for enhancing the effects of genetic markers in the diagnostics of hypophosphatasia disease. The simulation and real data analysis have demonstrated the effectiveness of our proposed integration methods, which can maximize genetic signals identified from the spinal cord and neocortex tissues, minimize the prediction error, and largely improve the prediction accuracy in risk prediction.

## 1. Introduction

Hypophosphatasia (HPP) is a rare inherited metabolic disorder, and its severe forms were estimated to affect 1 in 100,000 births in Canada and 1 in 300,000 in Europe, while moderate forms of HPP are 50 times more frequent [1]. US data suggest that HPP is more prevalent in white people than in black people [1]. The ethnic group with the highest reported incidence of HPP is the Mennonites in Manitoba, Canada, with 1 in 25 individuals carrying a tissue-nonspecific alkaline phosphatase (TNAP) mutation and around 1 in 25,000 newborns having lethal HPP [2]. In fact, HPP is caused by the deficiency in TNAP, and TNAP activity is essential for bone formation, mineralization, and differentiation of bone marrow stromal cells. Specifically, TNAP deficiency in bone and muscle progenitor cells results in mitochondrial hyperfunction and increased ATP levels, both of which greatly influence cell function and survival [3]. The clinical symptoms of HPP widely vary according to the patient’s age at the onset of the disorder. Less severe forms of HPP are often characterized by pain or fractures over time [4,5] due to poor bone mineralization. More severe and early onset cases of HPP present issues with muscle weakness, diminished motor coordination [3], and epileptic seizures [6]. These neurological manifestations are poorly characterized, especially in children. Therefore, the development of strategies to improve the neurological outcomes is urgently needed. One goal of this study is to propose a statistical model to accurately reveal the genetic mechanism underlying the neurological manifestations of HPP. In this study, the tissue samples come from *Alpl*+/+ (wild type) and *Alpl*−/− (Global TNAP knockout) mice to represent the phenotype of patients with infantile HPP in the human population [3]. We have collected two RNA-sequencing (RNA-seq) data from the spinal cord and neocortex tissues of a mouse model of infantile hypophosphatasia. The more important task is to develop a new method to integrate multiple RNA-seq data for discovering influential genes associated with the HPP disease and predicting the disease presence in subjects.

In 2008, RNA-seq was first introduced to the field of transcriptomics [7,8,9,10], and it has since greatly transformed the genetic study. Transcriptomics is the study of the complete set of RNA transcripts that are produced by the genome under specific conditions or in a specific cell. Studying transcript levels is essential to understand the translation process from information encoded in the genome to cellular functions. Consequently, transcript profiling is an important tool for predicting the presence of disease. RNA-seq offers several advantages over other types of transcriptome profiling, particularly single-cell RNA-seq which can identify novel transcripts not corresponding to an existing genomic sequence. Additionally, RNA-seq has a low background signal, and a large dynamic range of expression levels to detect transcripts, and it can precisely locate transcription boundaries down to a single-base resolution [11]. More practically, a large amount of RNA transcript is needed, but the cost of mapping whole genome is lower than other data, such as microarrays or cDNA sequencing [11].

While the applications of RNA-seq study have become widespread, this complex process faces a big challenge on how to properly analyze RNA-seq data [12]. There exist several pipelines and publications outlining best practices for the entire process from the extraction to the data analysis [12,13,14,15]. Many traditional statistical methods have been applied to RNA-seq data such as a likelihood ratio test [16] or negative binomial models [16,17]. With the fast growing of deep learning and machine learning in many different domains, deep learning models have recently been applied to RNA-seq data [18,19] with limited success since high-throughput sequencing data fights with the imbalance between a large number of genetic markers and a small number of samples.

As mentioned before, the number of genetic markers in a RNA-seq data is extremely greater than the number of samples, and this kind of data analysis faces a challenge on how to accurately explore the genetic effects from high-dimensional variants. Several frequentist methods have been developed to shrink genetic estimates and reduce bias, such as LASSO [20] and the shrunken centroids methods [21]. However, these methods tend to overestimate the genetic effects and include noisy signals. Fortunately, Bayesian approach produces estimates which are immune to estimation bias in a high-dimensional data analysis. In addition, an empirical Bayes (EB) method [22] can simplify the calculations of Bayesian approaches and help address the selection bias problem occurred in a large feature space. This method has been successfully applied to microarray data for a prostate cancer study [22,23]. Later, the EB method is extended to exome DNA sequencing data for predicting the risk of cardiovascular disease [24]. Recently, a weighted EB model is derived to incorporate multiple traits, a quantitative disease trait, and a binary disease status, in whole-genome DNA sequencing analysis for improving the genetic mechanism underlying a hypertension disease [25]. Here, with the collection of multiple RNA-seq data from the spinal cord and neocortex tissues of a mouse model, there remains a need to develop a new EB method which combines two RNA-seq data into a model for maximizing the effects of genetic markers, and effectively reducing the estimate bias in a high-dimensional data analysis.

In this study, we apply the EB method to the spinal cord and neocortex RNA-seq data, separately, for more accurately discovering the differentially expressed (DE) genes in the HPP disease study. More importantly, we further propose two integration methods, weighted gene approach and weighted *Z* method, to incorporate two RNA-seq data into a model for enhancing the effects of genetic variants in the diagnostics of HPP disease. We expect that the proposed integration methods can strengthen the genetic signals and improve the prediction performance, because some disease-causal genes may be differentially expressed at one specific tissue, not at both tissues. To test this conjecture, we compare the empirical Bayes method, which is applied to the single RNA-seq data, with two integration empirical Bayes methods jointly analyzing two RNA-seq data. The results will demonstrate the efficiency of our proposed integration methods.

This paper is outlined as follows. Section 2 discusses four EB methods. Section 3 proves the effectiveness of two integration methods. In the following Section 4, the specifics of a real RNA-seq data analysis are discussed, including a description of the datasets, distributions of the statistics, ranking of important features, and comparison of various methods. We conclude with a few remarks from the data analysis, and further discuss the limitations of current methods, and give some directions of future work in Section 5.

## 2. Methods

### 2.1. Step I: Gene Expression

**Figure 1:**
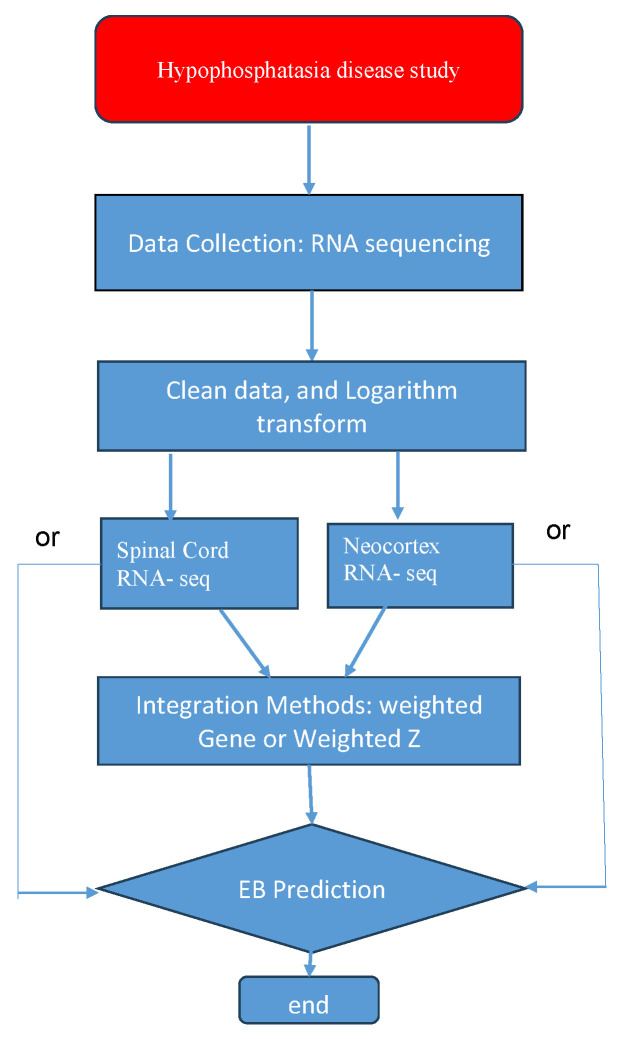
The workflow of this study.

The experimental procedures of this study were approved by the Institutional Animal Care and Use Committee (IACUC) of the University of Michigan (Protocol number: PRO00010860, date of approval: 22 August 2022). Here, two RNA-seq data are collected from spinal cord and neocortex tissues. Each data is composed of tens of thousands of genes collected from 16 mice. Those mice are classified into two categories, 8 mice are assigned to a wild type group and another 8 mice are in a knock-out group. A balanced design is applied here. The wild type is treated as a normal group, and the knock-out class is treated as a disease group. Specifically, two RNA-seq data share common samples, and the main discrepancy between two data may come from the assumption that combining DE genes detected from two tissues may strengthen the genetic signal in the diagnostics of HPP disease. Moreover, raw RNA-seq data is transformed via a binary logarithm function, so that its distributions based on the transformed data more closely resemble the normal distribution, which better aligns with the empirical Bayes method. The relevant workflow of study is summarized in Figure 1.

### 2.2. Step II: Empirical Bayes Prediction Rule for a Spinal Cord RNA-Sequencing Data

RNA-seq data collected from the spinal cord is critical to the central nervous system, and its analysis helps understand the genetic mechanism of the HPP disease. We assume that there are *N* genes $X_{p}=\left(X_{1|p},\ldots ,X_{N|p}\right)$ where $X_{p}$ is a n×N matrix measuring *N* log transformed genes of *n* subjects, and *p* is a label of spinal cord. The corresponding standardized gene matrix $W_{p}=\left(W_{1|p},\ldots ,W_{N|p}\right)$ is defined by an equation $W_{i|p}=\frac{X_{i|p}-\frac{\left(\mu_{i,1|p}+\mu_{i,2|p}\right)}{2}1_{n}}{\sigma_{i|p}}$, i = 1, ⋯, N where $X_{i|p}$ is one column of matrix $X_{p}$ measuring the $i^{th}$ transformed gene for all subjects, $1_{n}$ is a vector of size n containing ones, $\mu_{i,1|p}$ and $\mu_{i,2|p}$ denote the mean of the $i^{th}$ gene in the normal and disease groups, individually. $\sigma_{i|p}$ is the standard deviation of the $i^{th}$ gene, and *n* represents the total number of subjects consisting of $n_{1}$ controls (normal subjects) and $n_{2}$ cases (disease subjects) with $n_{1}=n_{2}=\frac{n}{2}$. Thus, the prediction rule is to classify subjects into the disease group if

(1) $$\sum_{i\leq N}\delta_{i|p}W_{i|p}>0$$

where the parameter $\delta_{i|p}=d_{0}\frac{\mu_{i,2|p}-\mu_{i,1|p}}{\sigma_{i|p}}$ ($d_{0}=\sqrt{\frac{n_{1}n_{2}}{n_{1}+n_{2}}}=\frac{\sqrt{n}}{2}$) measures the impact of the $i^{th}$ gene on the HPP disease. In this study, a balanced design is applied ($n_{1}=n_{2}$), then, a threshold zero is selected to define the decision boundary. In the above equation, a vector 0 is used to allocate subjects having large prediction values to a disease group.

In reality, $\sigma_{i|p}$ is often unknown, but it can be estimated by a statistic $s_{i|p}$ ($s_{i|p}=\sqrt{\frac{\left(n_{1}-1\right)s_{i,1|p}^{2}+\left(n_{2}-1\right)s_{i,2|p}^{2}}{n-2}}$), where $s_{i,1|p}^{2}$ and $s_{i,2|p}^{2}$ are sample variances of the $i^{th}$ gene in the normal class and disease group, respectively. Hence, the parameter $\delta_{i|p}$ will be estimated by

(2) $$t_{i|p}=d_{0}\frac{{\bar{X}}_{i,2|p}-{\bar{X}}_{i,1|p}}{s_{i|p}}∼t_{n-2}\left(\delta_{i},1\right)$$

Note that ${\bar{X}}_{i,k|p}=\frac{\sum_{j=1}^{n}X_{ij|p}I\left(Y_{j|p}=k-1\right)}{n_{k}}$ (*k* = 1 or 2) is the sample mean of the $i^{th}$ gene in the normal or disease group where $Y_{j|p}$ (= 0 or 1) is the *j*th subject’s disease status. To take the advantage of normal property, $t_{i|p}$ is converted into a normal variable $Z_{i|p}=\Phi^{-1}\left(P\left(T\leq t_{i|p}\right)\right)$, where $\Phi^{-1}$ is the inverse of the cumulative distribution function of standard normal and $P(T\leq t_{i|p})$ is a cumulative distribution function of *t* distribution with n−2 degrees of freedom. Thus, $Z_{i|p}$ becomes an estimate of $\delta_{i}$ when we analyze a spinal cord RNA-seq data, and it follows a normal distribution with mean $\delta_{i}$ and the standard deviation close to 1 [22] Remark F. The standardized gene $W_{i|p}$ is estimated by ${\hat{W}}_{i|p}=\frac{X_{i|p}-\frac{{\bar{X}}_{i,1|p}+{\bar{X}}_{i,2|p}}{2}1_{n}}{s_{i|p}}$. The prediction rule based on $Z_{i|p}$ will be defined below,

$$\sum_{i\leq N}Z_{i|p}{\hat{W}}_{i|p}>0$$

However, the estimate $Z_{i|p}$ may result in a selection bias, wherein genetic effects are often overestimated in the above prediction rule. It is known that Bayesian methods may provide less biased estimates [26,27], and previous studies [28,29] have shown that the marginal density function of statistic $Z_{i|p}$ is capable of deriving the Bayesian estimate without knowing the prior of $\delta_{i}$. For any pair (*Z*, δ), the Bayesian estimate $\hat{\delta }$ is obtained by the first derivative of the logarithm of the marginal density of *Z* [22],

$$\begin{array}{c} Z|\delta ∼N(\delta ,1)\\ \hat{l_{f}}\left(z\right)=log\left(\hat{f}\left(z\right)\right)\\ \hat{\delta }=z+s^{2}{\hat{l_{f}}}^{\prime }\left(z\right)=\hat{E}\left(\delta |z\right) \end{array}$$

where $\hat{l_{f}}\left(.\right)$ estimates the logarithm of the marginal density of *Z*, ${\hat{l_{f}}}^{\prime }\left(z\right)$ is its first derivative function, and $s^{2}$ (sample variance) is numerically calculated. Basically, the empirical Bayes method provides a numerical approximation of the Bayes estimates [22].

Finally, a subset of genes with large EB estimates denoted as ${\hat{\delta }}_{i|p}$ define the following prediction rule:

(3) $$\sum_{i\in I_{1}}{\hat{\delta }}_{i|p}{\hat{W}}_{i|p}>0$$

where a set $I_{1}$ collects all potential genes with strong EB estimates (${\hat{\delta }}_{i|p}$, $i\in I_{1}$) in a spinal cord RNA-seq analysis.

### 2.3. Step III: Empirical Bayes Prediction Rule for a Neocortex RNA-Sequencing Data

The second RNA-seq data is collected from the brain neocortex of the mouse, and it is also used to explore the genetic mechanism of HPP disease. Here, the feature dimension of the neocortex RNA-seq data is the same as that of the above spinal cord data. For *N* genes, $X_{c}=\left(X_{1|c},\ldots ,X_{N|c}\right)$ denotes all genes measured from the neocortex tissue, and the corresponding standardized gene matrix $W_{c}=\left(W_{1|c},\ldots ,W_{N|c}\right)$ is calculated by $W_{i|c}=\left[X_{i|c}-\frac{\left(\mu_{i,1|c}+\mu_{i,2|c}\right)}{2}1_{n}\right]/\sigma_{i|c}$, i = 1, …, N, where *c* is a label of the neocortex, $\mu_{i,1|c}$ and $\mu_{i,2|c}$ denote the mean of the $i^{th}$ gene in the normal and disease groups, individually, $\sigma_{i|c}$ denotes the standard deviation of the $i^{th}$ gene, and *n* represents the total number of subjects. Since two RNA-seq data share the common samples, *n*, $n_{1}$, and $n_{2}$ can be defined in the same way. Thus, the prediction rule is to classify subjects to the disease group if

$$\sum_{i\leq N}\delta_{i}W_{i|c}>0$$

where the parameter $\delta_{i}=d_{0}\frac{\mu_{i,2|c}-\mu_{i,1|c}}{\sigma_{i|c}}$ assesses the impact of the $i^{th}$ gene on the disease when we analyze neocortex RNA-seq data. Since an equal number of samples are assigned to the normal and disease groups, a balanced design may select a zero threshold to determine the decision boundary.

Similarly, an estimator $t_{i|c}$ of $\delta_{i}$ is calculated below:

$$t_{i|c}=d_{0}\frac{{\bar{X}}_{i,2|c}-{\bar{X}}_{i,1|c}}{s_{i|c}}∼t_{n-2}\left(\delta_{i},1\right)$$

Note that this statistic $t_{i|c}$ is also converted into a normal variable $Z_{i|c}=\Phi^{-1}\left(P\left(T\leq t_{i|c}\right)\right)$, which has a selection bias in practice. We shrink $Z_{i|c}$ to obtain the relevant EB estimator ${\hat{\delta }}_{i|c}$ for the $i^{th}$ gene (i=1,...,N). The final prediction rule of a neocortex RNA-seq data analysis is defined as

(4) $$\sum_{i\in I_{2}}{\hat{\delta }}_{i|c}{\hat{W}}_{i|c}>0$$

where ${\hat{W}}_{i|c}$ is the $i^{th}$ sample standardized gene vector calculated from the neocortex data, and its formula is similar to that of Section 2.2, and a set $I_{2}$ collects all genes with important EB estimates (${\hat{\delta }}_{i|c}$, $i\in I_{2}$).

### 2.4. Step IV: Empirical Bayes Prediction Rule When Integrating the Spinal Cord and Neocortex RNA-Seq Data

#### 2.4.1. Weighted Gene Approach

Since two RNA-seq data are collected from spinal cord and neocortex tissues, we expect that combining the genetic signals from multiple RNA-seq data may improve the genetic effects in the diagnostics of HPP disease. The idea of weighted gene method was first developed in the empirical Bayes model by integrating the synonymous gene and non-synonymous gene together for improving the cardiovascular disease prediction performance in an exome DNA sequencing study [24]. Here, we extend this idea to combine two RNA-seq data acquired from the spinal cord and neocortex tissues, and it may maximize the genetic signals.

For all subjects, $X_{p}$ and $X_{c}$ denote *N* genes of the spinal cord and neocortex, individually, where *p* represents spinal cord, and *c* denotes neocortex. While we analyze two RNA-seq data, a weight ($w_{i}$) measuring the relative importance of the $i^{th}$ gene effect detected from the spinal cord data compared to that from the neocortex data is calculated by

(5) $$w_{i}=\frac{-\log(p_{i|p})}{-\log\left(p_{i|p}\right)-\log\left(p_{i|c}\right)}\;\mathrm{for}\;i=1,\ldots ,N$$

where $p_{i|p}$ and $p_{i|c}$ are *p*-values calculated from a simple logistic regression model which detects the $i^{th}$ gene effect on the HPP disease corresponding to the spinal cord data and neocortex data, individually. A larger weight suggests that the genetic effect identified from the spinal cord may have a relatively stronger association with the disease than that from the neocortex. A new weighted gene ($X_{i}^{*}$) will make use of this weight to combine the genetic effects from two RNA-seq data, and it is defined below:

(6) $$X_{i}^{*}=w_{i}X_{i|p}+\left(1-w_{i}\right)X_{i|c}\;\mathrm{for}\;i=1,\ldots ,N$$

These *N* weighted genes $\left(X_{1}^{*},\ldots ,X_{N}^{*}\right)$ are capable of maximizing the genetic effects detected from the spinal cord and neocortex tissues. The relative empirical Bayes prediction rule will classify subjects to the disease if

(7) $$\sum_{i\leq N}\delta_{i}W_{i}^{*}>0$$

Note that $\delta_{i}$ measures the genetic effect of the $i^{th}$ standardized weighted gene, where $W_{i}^{*}=\frac{X_{i}^{*}-\frac{\left(\mu_{i,1}^{*}+\mu_{i,2}^{*}\right)}{2}1_{n}}{\sigma_{i}^{*}}(i=1,\ldots ,N$) is the $i^{th}$ standardized weighted gene which is defined similarly as in the aforementioned section. Similarly, $\mu_{i,1}^{*}$ and $\mu_{i,2}^{*}$ are the mean values of the $i^{th}$ weighted gene corresponding to the normal group and the disease group, and $\sigma_{i}^{*}$ is the standard deviation of the $i^{th}$ weighted gene. As before, the statistic ($t_{i}^{*}$) is calculated to estimate the parameter $\delta_{i}$ which reflects the effect of the $i^{th}$ weighted gene.

$$t_{i}^{*}=d_{0}\frac{{\bar{X}}_{i,2}^{*}-{\bar{X}}_{i,1}^{*}}{s_{i}^{*}}∼t_{n-2}\left(\delta_{i},1\right)$$

$$Z_{i}^{*}=\Phi^{-1}\left(P\left(T\leq t_{i}^{*}\right)\right)$$

where ${\bar{X}}_{i,1}^{*}$ and ${\bar{X}}_{i,2}^{*}$ are sample means of the $i^{th}$ weighted gene in the normal class and disease class, respectively, $s_{i}^{*}$ is the pooled sample standard deviation of the $i^{th}$ weighted gene, and $d_{0}=\sqrt{\frac{n_{1}*n_{2}}{n_{1}+n_{2}}}$. A statistic $t_{i}^{*}$ is converted into an estimator $Z_{i}^{*}$ through an inverse standard normal function $\Phi^{-1}$ and a cumulative distribution function $P(T\leq t_{i}^{*})$.

The normal property of $Z_{i}^{*}$ guarantees the effectiveness of parameter $\delta_{i}$ estimation. To avoid the selection bias, the empirical Bayes method will shrink the estimator $Z_{i}^{*}$ to an EB estimate ${\hat{\delta }}_{i}$. The final prediction rule will be based on all important EB estimates,

$$\sum_{i\in I_{3}}{\hat{\delta }}_{i}{\hat{W}}_{i}^{*}>0$$

where a set $I_{3}$ collects all standardized weighted genes having strong EB estimates, ${\hat{W}}_{i}^{*}$ is the estimate of the $i^{th}$ standardized weighted gene combining spinal cord and neocortex genetic signals, and its standardized formula is defined similarly as in the previous section.

#### 2.4.2. Weighted *Z* Method

The principle of weighted gene method is to calculate a weight ($w_{i}$) for each gene, and it enlarges the genetic signal from two RNA-seq data. An alternative way for strengthening the genetic signal is to calculate a weighted *Z* score, which combines a *Z* statistic detected from the spinal cord data with that from the neocortex data. We expect to increase the genetic effects when scanning two RNA-seq data.

$Z_{i|p}$ defined in Section 2.2 is a genetic estimate of the $i^{th}$ gene, and this statistic reflects the importance of the $i^{th}$ gene identified from spinal cord data. Similarly, $Z_{i|c}$ defined in Section 2.3 is also an estimate of the $i^{th}$ gene, and it explores the genetic effect calculated from the neocortex data. A weight $zw_{i}$ is defined below:

(8) $$zw_{i}=\frac{|Z_{i|p}|}{|Z_{i|p}\left|+\right|Z_{i|c}|}\;\mathrm{for}\;i=1,\ldots ,N$$

where $|Z_{i|p}|$ and $|Z_{i|c}|$ are absolute values of statistics calculated from the spinal cord data and neocortex data, respectively. For a fixed sample data, the weight $zw_{i}$ reflects the relative importance of the $i^{th}$ gene effect detected from a spinal cord data compared to that from a neocortex data. A larger weight indicates that the $i^{th}$ gene detected from the spinal cord shows a stronger signal than that from the neocortex. To better capture the genetic effect from two RNA-seq data, a weighted *Z* statistic is calculated below:

(9) $$Z_{i}^{w}=zw_{i}Z_{i|p}+\left(1-zw_{i}\right)Z_{i|c}$$

According to Section 2.2 and Section 2.3, $Z_{i|p}$ and $Z_{i|c}$ follow the normal distribution with mean $\delta_{i}$ and variance close to 1, respectively. The weighted $Z_{i}^{w}$ is a function of two variables $Z_{i|p}$ and $Z_{i|c}$. While data is fixed, this new statistic $Z_{i}^{w}$ approximates a normal distribution with mean $\delta_{i}$ and the standard deviation $s_{Z_{i}^{w}}$ ($s_{Z_{i}^{w}}=\sqrt{zw_{i}^{2}+{\left(1-zw_{i}\right)}^{2}+2zw_{i}\left(1-zw_{i}\right)\rho }$), where ρ is the correlation coefficient between two statistics $Z_{p}$ and $Z_{c}$ calculated from the spinal cord and neocortex data, and it will help calculate the standard deviation of the statistic $Z_{i}^{w}$. In real application, this new statistic $Z_{i}^{w}$ reflects the genetic effect of the $i^{th}$ gene after combining two statistics $Z_{i|p}$ and $Z_{i|c}$. Then, we apply the empirical Bayes method to shrink this statistic ($Z_{i}^{w}$), and the relevant EB estimate $\hat{\delta_{i}}$ is estimated to avoid selection bias. The final prediction rule will be determined by all important EB estimates.

## 3. Simulation

### Simulation Analysis

**Figure 2:**
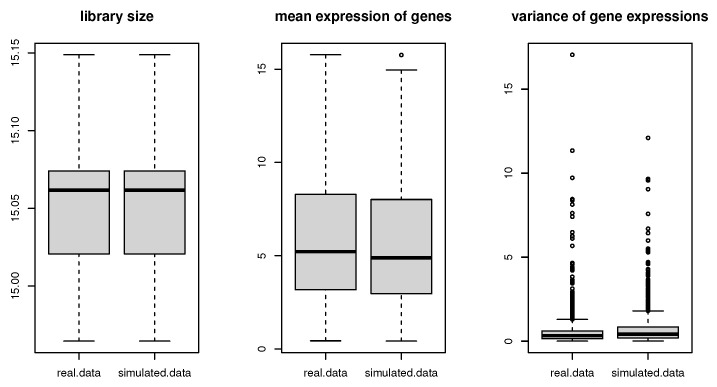
Simulation of spinal cord RNA-seq data.

**Figure 3:**
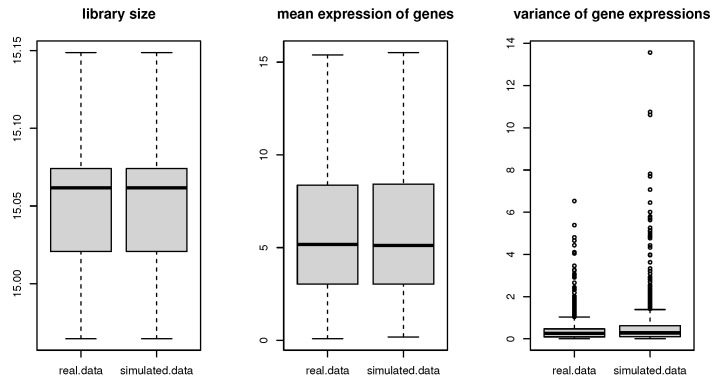
Simulation of neocortex RNA-seq data.

To assess the performances of our proposed methods, a semi-parametric simulation method (SPsimSeq: [30]) is applied to generate two RNA-seq data corresponding to spinal cord and neocortex RNA-seq data. This simulation method requires to input raw count data, then, it is designed to maximally retain the characteristics of real RNA-seq data. In particular, two simulation data are capable of capturing the gene-wise distributions and the between-genes correlation structure of real source data. In the simulation scenario, 3000 genes and 16 samples are simulated. Specifically, around 2% of genes (65 genes) are selected to be DE genes which are important to HPP disease, and the remaining genes are null genes. A total of 16 samples are allocated to two classes, then, eight subjects are assigned to disease and eight subjects are assigned to normal. Figure 2 and Figure 3 compare the variability and distribution of mean expression levels between simulation data and real data. It clearly illustrates that both simulation data have retain major characteristics of real spinal cord and neocortex RNA-seq data.

Feature selection is a key step in the risk prediction, where genes are ranked by their importance to the disease. All discussed methods are compared by their discoveries of true DE genes. We expect more DE genes selected by our proposed methods to possess larger effects and occupy top positions in the list. Table 1 summarizes the number of DE genes among top 100 or 200 genes identified by each method. The more the discovery of DE genes is, the better the corresponding method is. The first two methods (Spinal and Cortex) apply the empirical Bayes model to single spinal cord simulation data and neocortex data, respectively (Table 1). This shows that single spinal cord data analysis can discover 21 true DE genes among top 100/200 genes, and the neocortex data analysis only identifies five DE genes among top 100/200 genes. These two simulation results are consistent with the findings of real data analysis. Compared to spinal cord data, neocortex data shows poor genetic singles. Table 1 also summarizes two integration methods’ results, such as weighted gene approach which detects 53 and 58 DE genes among top 100 genes and top 200 genes, respectively. This method tries to maximize the genetic signals from two RNA-seq data, thus, 53 selected DE genes consist of 44 spinal cord DE genes and nine neocortex DE genes, and 58 DE genes include 49 spinal cord DE genes and nine neocortex DE genes. The alternative integration method, weighted *Z*, can even detect a larger number of true genes, such as 56 DE genes among top 100 genes, and 61 DE genes among top 200 genes. This finding is also consistent with the real data analysis, where weighted *Z* performs better in the areas of prediction error and accuracy. In addition, we also display the details of top 30 genes ranked by various methods (Table 2). In particular, yellow color highlights the spinal cord DE genes, and cyan color denotes the neocortex DE genes. This illustrates that two integration methods are capable of strengthening the genetic signals from two simulation RNA-seq data.

We further compare the prediction errors of various methods. Both simulation data are split into two sets, a training dataset (50% samples) and a test dataset (50% samples). Each proposed model is fit into a training set, then its model estimate is applied to a test set to calculate the prediction error. We repeat this cross-validation (CV) procedure five times to calculate the average test error. Table 3 summarizes the error rate and its standard error of each method. The results demonstrate that two integration methods perform best, like weighted *Z* has the smallest error rate (0.16), and weighted gene approach (0.225), followed by single spinal cord data analysis (0.275), and single neocortex data analysis (0.3). In general, the simulation study helps understand RNA-seq data properties. (1) Two simulation data retain major characteristics of real spinal cord and neocortex RNA-seq data. (2) Two integration methods, weighted gene and weighted *Z*, detect a larger number of DE genes and receive a smaller prediction error, compared to the single spinal cord data analysis or single neocortex data analysis. (3) If we focus on two single data analyses (spinal cord and neocortex) compared to each other, the spinal cord data makes it easier to detect DE genes. It seems that the stronger effects of genes are detected from the spinal cord tissue. (4) The performances of simulation data are generally consistent with those of real data.

## 4. Real Application

### Data Analysis

Hypophosphatasia is a rare inheritable disorder caused by TNAP deficiency. Previous studies have demonstrated that TNAP deficiency results in sensorimotor dysfunction [3]. Here, two RNA-seq data acquired from the brain neocortex and spinal cord of Alpl+/+ (wild-type) and Alpl−/− (Global TNAP knockout) mice have been collected. This transgenic mouse model of infantile HPP represents the more severe phenotype of HPP in the human population. We expect that RNA transcriptional profile data near candidate genes may enrich the effects of genetic variants that fall within a sequence data, and any new findings will help better understand the mechanisms of pediatric brain injuries during brain development. In this study, both RNA-seq data include 36,500 gene markers and 16 samples. According to one covariate, gender, male and female mice are equally allocated to normal and disease groups, as seen in Appendix A Table A5. Noticeably, the number of genes is much larger than the number of samples. Consequently, both spinal cord and neocortex data are cleaned to further reduce their feature dimensionality. The initiate analysis, providing $\log_{2}$ fold-change, log fold-change standard error, and *t* statistic, serves as reference for cleaning the data. The criteria suggest that few genes having a small difference in fold change between the wild-type and knock-out mice, and having an extremely smaller standard error, should be removed. These genes may easily provide the false positive signals and increase the detection noise when we scan all genes in an RNA-seq study. After cleaning the data, 28,426 genes and 16 samples are included in two RNA-seq datasets.

**Figure 4:**
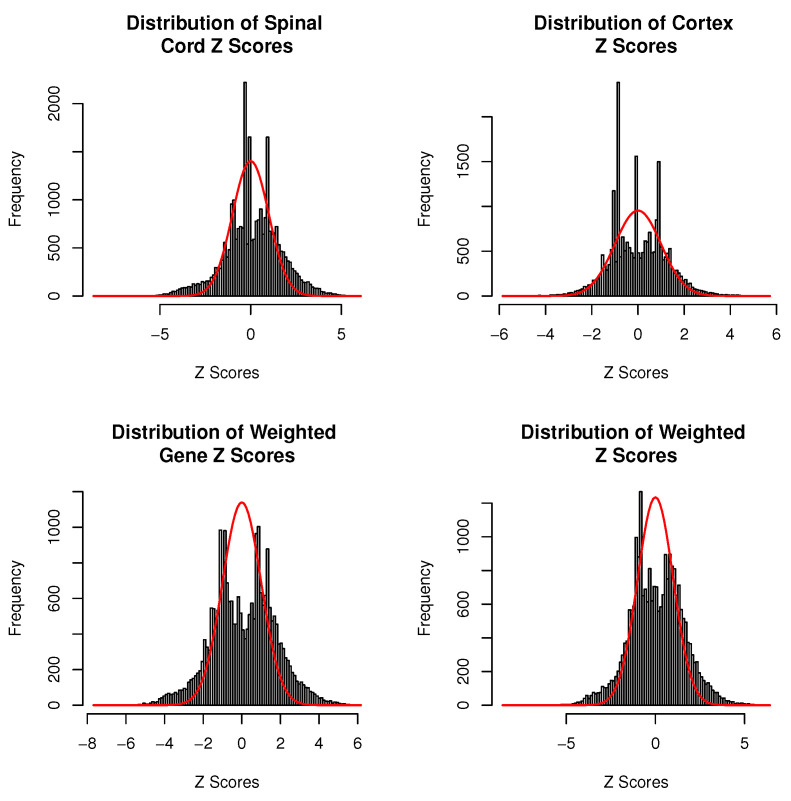
Distribution of *Z* statistics generated by a spinal cord data, a neocortex data, and two integration methods.

It is known that the statistic *Z* estimates the genetic effect for each gene, and its normal property will assure the successful application of the empirical Bayes method. Figure 4 summarizes four histograms of all genes’ *Z* scores calculated from the spinal cord, neocortex, weighted gene, and weighted *Z* methods, respectively. Specifically, the red color line in each subplot represents the standard normal density curve. Compared to the integration methods, the first two subplots corresponding to the spinal cord and neocortex methods show that *Z* statistics have a small tail, which makes the detection of novel genes more challenging. One possible reason is that these two analyses only consider single RNA-seq data information. If we further compare the subplots between the spinal cord and the neocortex, it may be revealed that the spinal cord analysis seems to have a heavier tail, and a good normal property, which may result in a better performance on genetic detections in the RNA-seq study. Conversely, two integration methods, weighted gene and weighted *Z* methods, display the heavier tails on both sides (negative/positive), which suggests we may easily detect more causal genes which are associated to the HPP disease.

One key step in the empirical Bayes method [22] is to rank the selected genes (features). All methods rank the most important genes by their respective empirical Bayes estimates. The top gene lists of four methods (spinal cord, neocortex, weighted gene, and weighted *Z*) are summarized. It is known that the genetic effects from the spinal cord and brain neocortex are important to the central nervous system. When we integrate two RNA-seq data, we expect that the combined method will more efficiently search for the most important genetic markers, and its performance will be better than the analysis result based on a single RNA-seq data. Most importantly, HPP is characterized by the reduced serum alkaline phosphatase (ALP), and its molecular diagnosis is established by identifying the loss-of-function ALPL variants [31], which is the most important gene highly related to HPP disease. In this study, Table 4 displays top 10 most important genes from a spinal cord data analysis, and it successfully detects the Alpl gene (position: No. 4). Table 4 also summarizes top 10 genes based on a neocortex data analysis, and it also detects Alpl, but it is ranked much lower at No. 26. It is interesting to see that another gene, Cirbp, is ranked much higher (position: No. 2). This gene (Cirbp) is related to the cold inducible RNA binding protein, and may be important in the diagnosis of the HPP disease. Since two RNA-seq data are collected, it is capable of proposing two integration methods described in Section 2.4.1 and Section 2.4.2 to strengthen as many genetic signals as possible. In particular, Table 5 summarizes top 10 gene list from the weighted gene method, and its list includes Alpl (Position: No. 6) and Cirbp (Position: No. 9). It seems that the weighted gene method can identify these two genes among top 10 genes by maximizing the genetic effects from two RNA-seq datasets. Additionally, more genes, namely GM13230, zfp990, Tmprss11d, and Eno1b, detected by the single spinal cord data analysis, are also observed in the top four gene positions. The second integration method, weighted *Z* method, displays its top 10 genes in Table 5. It also detects two genes (Alpl and Cirbp), but two genes (Cirbp and Fkbp5) identified by the single neocortex data analysis are ranked at the front, followed by the genes Alpl, GM13230, zfp990 and Eno1b detected by the single spinal cord data analysis. This may suggest that weighted *Z* method provides an alternative way to maximize the genetic effects from two RNA-seq data. When we extend the top 10 genes to the top 40 genes, it is better to see that more top-ranked genes detected by a spinal cord data analysis or a neocortex data analysis are selected by the integration methods. The relevant top 40 gene lists are summarized in Appendix A (Table A1, Table A2, Table A3 and Table A4). Generally, the most important HPP disease causal gene, Alpl, is successfully detected by four methods, especially the integration methods which are capable of maximizing multiple gene signals from a spinal cord data and a neocortex RNA-seq data. HPP is a complex disease, and its diagnosis is based on the collective actions of multiple genes. Identifying more genes will help us better understand their biological effects in the diagnosis of HPP disease.

The identifications of most important genes complete the feature selection procedure. The next step is to evaluate the risk prediction based on the selected candidate genes. We split samples into a train and a test set. Since the sample size is rather small, 50% of the subjects are randomly assigned to a train set, and the remaining goes to a test group. This cross-validation is repeated five times to calculate the average test error. Table 6 summarizes and compares the prediction errors of four methods. Specifically, the prediction error of a neocortex data analysis gives the largest error rate (0.15). In fact, this result is consistent with its poor performance in the *Z* histogram. While the EB prediction rule applies to a spinal cord data, it performs slightly better and a smaller prediction error (0.125) is obtained. The proposed integration methods, weighted gene and weighted *Z* methods, expect to better search the most important genes for risk prediction. In particular, the error rate of weighted gene method is 0.1, which is smaller than the error rates based on the single spinal cord and the neocortex data analysis. Weighted *Z* method combines two *Z* statistics detected from two RNA-seq datasets to select the causal genes, and those selected genes will be separately applied to spinal cord and neocortex data to compute the test error rates. The final error is based on the average value of two data results, and it is around 0.0625, which shows the minimum prediction error. In summary, the integration methods, weighted *Z* and weighted gene methods, provide the smaller prediction error rates, and these two methods may better explore the genetic effects in the risk prediction of HPP disease.

**Figure 5:**
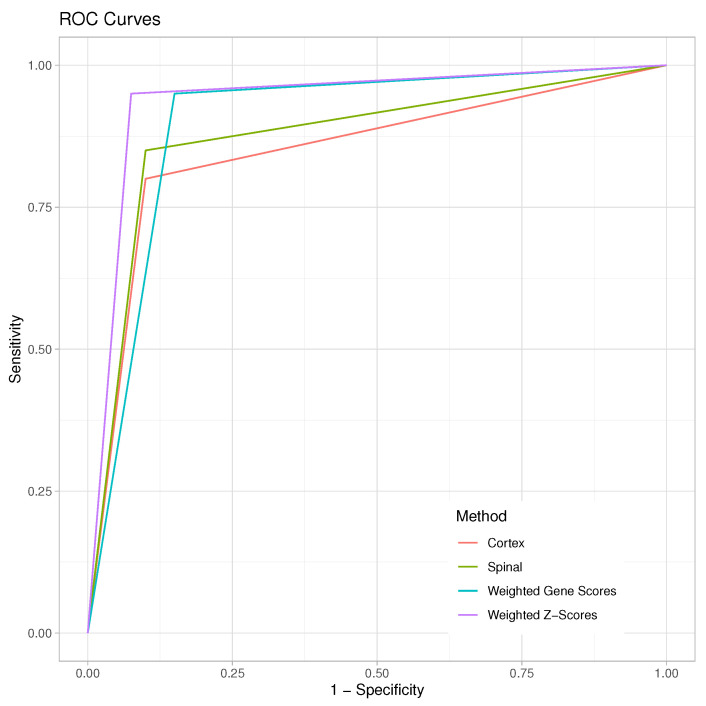
ROC curves of various methods.

We also calculate the area under the receiver operating characteristic (ROC) curve (AUC) to compare the prediction accuracy among four different methods. Table 7 compares the prediction accuracy across four methods. Specifically, the neocortex data analysis gives the smallest prediction accuracy (AUC: 85%), followed by the spinal cord data analysis, where its AUC achieves 87.5%; then, weighted gene approach achieves an AUC of 90%, and weighted *Z* method has the highest prediction accuracy (AUC: 93.75%). This analysis reveals the importance of empirical Bayes estimates in developing a large-scale risk prediction model, and two integration methods further improve the prediction accuracy while jointly analyzing two RNA-seq data. Figure 5 illustrates the comparison of ROCs across four methods under the cross-validation procedure. This figure demonstrates that two integration methods outperform the individual RNA-seq data analysis in terms of prediction accuracy. The results imply that our proposed methods are efficient prediction tools for a large-scale RNA-seq data analysis.

## 5. Discussion

Hypophosphatasia is a rare inherited metabolic disorder caused by tissue-nonspecific alkaline phosphatase deficiency which influences cell function and survival [3]. It manifests as a variety of clinical symptoms where more severe and early onset cases are often characterized by neurological symptoms, such as sensorimotor dysfunction [3] or epileptic seizures [6]. However, these neurological manifestations are poorly characterized, particularly in children. RNA-seq data offer a high-resolution and highly accurate transcript profile, which is an important tool for predicting disease risk. In this study, two RNA-seq data are acquired from spinal cord and neocortex tissues of a mouse model to explore the genetic mechanism underlying the diagnostics of HPP disease. Typically, RNA-seq data result in a large number of predictors and a limited number of samples, which challenges many traditional statistical methods because these methods may easily overestimate the genetic effects due to a selection bias. A method such as empirical Bayes [22] attempts to mitigate this problem by shrinking the estimates in the gene expression study. Therefore, we propose two integration methods, weighted gene and weighted *Z*, to jointly analyze multiple RNA-seq data for maximizing the genetic signals in an empirical Bayes prediction rule. This study will help develop age-specific strategies and improve neurological outcomes in patients.

This project focuses on the discoveries of differentially expressed genes from multiple RNA-seq data, but HPP disease may be caused by the complex relationships between genetic factors and non-genetic factors. In real applications, genes often interact with each other or act with the environment factors, therefore, research adding gene–gene and gene–environment interactions seems to be beneficial. More importantly, the differential expressed genes in mammals play an important role in controlling brain growth and development, especially for children. The malfunction of genes at any developmental stage could lead to substantially abnormal characters such as genetic disorders. As a highly complex process, changes in gene expression can redirect its developmental trajectory to better adapt to environmental conditions. For this reason, incorporating such information into the empirical Bayes model should provide more information about the genetic architecture of a dynamic developmental trait.

The long-term goal is to discover the interaction between genetic transcriptome and metabolic markers underlying the diagnostics of HPP disease. In addition to RNA-seq data, we have collected metabolome data from the spinal cord and neocortex tissues of mice. These multiple omics data will help develop the age-specific and pathway-specific personalized/targeted treatments. Since metabolites data directly reflect the changes in disease phenotype and ensuing effects of post-translational modifications [32], it is increasingly collected for biomarker discovery. Current integration approaches fail to capture complex or indirect relationship between transcripts and metabolites, furthermore, the pathway methods are limited to metabolites, and only a small fraction of metabolites have been mapped to it. A promising method IntLIM [33] integrates trancriptomic and metabolomic data via a linear model, which provides a way to discover different performances of the gene–metabolite interaction between normal and disease groups. Thus, our future work is going to integrate and augment different omics data, such as RNA-seq data and metabolomics data, for recognizing the complex architecture of markers identified from multiple omics data during the development of HPP disease.

The most difficult obstacles in this study is the small number of samples (n=16). Due to the input uncertainty, our analysis may make the genetic estimates not very stable. Specifically, while calculating the average prediction error, it is impossible to strictly follow the cross-validation procedure. We have to split raw data into 50-50 subsets, and repeat it five times. This procedure leads to a potential issue (a kind of false positive) that five training sets/five test data share a large proportion of common samples. However, it is rather difficult to enlarge the sample size due to the budget limitation. For this reason, we plan to work on a new theoretical method, distribution robust optimization (DRO) approach, to obtain steady genetic estimates. DRO method [34] is attractive because it combines features of robust optimization method and stochastic optimization method to inherit the benefits of both methods. In general, DRO can overcome the conservative estimates of the robust optimization method without the exact distribution required in stochastic optimization. Thus, a new EB model incorporating the principle of DRO method may take the advantage of its theoretical properties and computational advantage, which will benefit from both the discovery of DE genes and the genetic interaction between metabolomic markers and DE genes when analyzing a small number of samples.

## Figures and Tables

**Table 1:** Number of true genes in the top 100/200 genes.

Simulation	Top Genes	Spinal	Cortex	Weighted Genes	Weighted Z
Simulation data	100	21	5	53 (S = 44, C = 9)	56 (S = 47, C = 9)
	200	21	5	58 (S = 49, C = 9)	61 (S = 51, C = 10)

Note: Spinal: EB method is applied to a single spinal cord simulation data. Cortex: EB method is applied to a single neocortex simulation data. Weighted Genes (*p*): the weighted gene approach is jointly applied to two simulation data. Weighted Z: the weighted Z method is jointly applied to two simulation data. Row 1 is used to calculate how many true DE genes are among top 100 genes detected by various methods. Row 2 is used to calculate how many true DE genes are among top 200 genes identified by various methods.

**Table 2:** Top 30 genes detected by 4 methods.

Ranking	Spinal	Cortex	Weighted Genes	Weighted Z
1	Glul	Gm44677	Klhl6	Gm43364
2	Tmem109	Lpl	Jpt2	Gm36947
3	Zic4	Sema4b	Gng11	Gm20939
4	Gm43300	Spint2	Aqp4	Gm13230
5	Asprv1	Inpp5f	Rmi2	Rex2
6	Rbm24	Gucy2f	Inpp5f	Gm3608
7	Per1	Edem1	Gm3608	Eno1b
8	Gm9768	2810029C07Rik	Mzb1	Idi1
9	Pmaip1	Phf1	Hemgn	Aplnr
10	Tmem64	Ncapg2	Cideb	Klhl6
11	Zfp687	Ccser2	Prr11	Pif1
12	Smim3	Slco3a1	Gm36947	Rmi2
13	Dsp	Dgcr8	Uhrf1	Ska1
14	Gm37567	Gnptab	Kntc1	Gng11
15	Mest	Ccdc166	Idi1	Igsf1
16	Fam107a	AI197445	Aplnr	Mki67
17	C2cd4a	Stil	Btnl10	Kntc1
18	Cdkn1a	Camk2d	Nxnl2	Alpl
19	St6galnac2	Mfsd10	Alpl	Hemgn
20	Sp6	Nxnl2	Ska1	Knl1
21	Ldoc1	Gm3203	Igsf1	Rdh5
22	Cyp2d22	Adam15	Gm43364	Aqp4
23	Rtbdn	Gm43364	Mki67	Mzb1
24	Mertk	Nup160	Cdc6	Jpt2
25	4930481A15Rik	Esyt3	Has2	Has2
26	Znf41-ps	Gm11427	Kcnj5	Cideb
27	Gm37310	Cpz	Gm20939	Nxnl2
28	Cd209f	Bub1	Bub1	Prr11
29	Gm37885	Rex2	Rex2	Btnl10
30	Gm4949	Eno1b	Eno1b	Cdc6

Note: yellow color denotes the spinal cord DE genes. Cyan color represents the neocortex DE genes.

**Table 3:** Prediction errors of various methods.

Simulation	Spinal	Cortex	Weighted Genes	Weighted Z
Error	0.275	0.3	0.225	0.1625
SD	0.285	0.068	0.185	0.0948

Note: Error denotes the average prediction error; SD is a standard deviation of prediction error; Spinal cord: a spinal cord RNA-seq data analysis; Cortex: a neocortex RNA-seq data analysis; Weighted Genes (*p*): weighted gene method; Weighted *Z*: weighted *Z* method.

**Table 4:** Top 10 gene list for a spinal cord data analysis and a neocortex data analysis.

Spinal Cord		Neocortex	
Ranking	Gene	Ranking	Gene
1	Gm13230	1	Gm49327
2	Zfp990	2	Cirbp
3	Eno1b	3	Txnip
4	Alpl	4	Igfbp3
5	Tmprss11d	5	Pla2g3
6	Ubiad1	6	Cbln3
7	Gm29367	7	Fkbp5
8	Rbm3	8	Gm20521
9	Sult1a1	9	Col23a1
10	Miip	10	Gm30003

Spinal Cord: empirical Bayes estimates for spinal cord RNA-seq data; Neocortex: empirical Bayes estimates for neocortex RNA-seq data.

**Table 5:** Top 10 gene list for two integration methods, weighted gene and weighted *Z*.

Weighted Gene		Weighted *Z*	
Ranking	Gene	Ranking	Gene
1	Gm13230	1	Cirbp
2	Zfp990	2	Slc25a34
3	Tmprss11d	3	Miip
4	Eno1b	4	Fkbp5
5	Hsph1	5	Gm29367
6	Alpl	6	Sult1a1
7	Masp2	7	Gm13230
8	Gm29367	8	Zfp990
9	Cirbp	9	Eno1b
10	Slc25a34	10	Alpl

Note: Top 10 genes based on two integration methods; Weighted Gene: the weighted gene method; Weighted *Z*: the weighted *Z* method.

**Table 6:** Average prediction error of four methods.

	Spinal Cord	Neocortex	Weighted Gene	Weighted *Z*
Error	0.125	0.1500	0.1000	0.0625
SD	0.1531	0.0559	0.1630	0.0765

Note: Error denotes the average prediction error; SD is a standard deviation of prediction error; Spinal cord: a spinal cord RNA-seq data analysis; Neocortex: a neocortex RNA-seq data analysis; Weighted Gene: the weighted gene method; Weighted *Z*: the weighted *Z* method.

**Table 7:** The prediction accuracy of four methods.

Method	AUC
Spinal	87.5%
Neocortex	85%
Weighted Gene	90%
Weighted Z	93.75%

AUC: is the area under the receiver operating characteristic. Spinal cord: a spinal cord RNA-seq data analysis; Neocortex: a neocortex RNA-seq data analysis; Weighted Gene: the weighted gene method; Weighted *Z*: the weighted *Z* method.

## Data Availability

The datasets and software codes are available online https://drive.google.com/drive/folders/1M9iU_2lG24329jiGFZI81UFFj8LtDlUZ?usp=sharing (accessed on 23 February 2024).

## Abbreviations

The following abbreviations are used in this manuscript:

RNA-seq	RNA-sequencing data
HPP	Hypophosphatasia
TNAP	Tissue-nonspecific alkaline phosphatase
EB	Empirical Bayes
DE	Differentially expressed

## Appendix A

**Table A1:** Top 40 most important genes for the EB classifier using spinal cord data.

Ranking	Gene	Ranking	Gene
1	Gm13230	21	Masp2
2	Zfp990	22	Plcd3
3	Eno1b	23	Kirrel2
4	Alpl	24	Mt2
5	Tmprss11d	25	Map3k6
6	Ubiad1	26	Zfp992
7	Gm29367	27	Elf5
8	Rbm3	28	Gm44421
9	Sult1a1	29	Smim3
10	Miip	30	Pxmp2
11	Tnfrsf8	31	Deaf1
12	Hsph1	32	Arrdc1
13	Klf15	33	Acer2
14	Tekt4	34	Fkbp5
15	Gm17244	35	Gm38246
16	Slc25a34	36	Paqr5
17	Gstm1	37	Aacs
18	Cirbp	38	Prodh
19	Gtdc1	39	Cnmd
20	1810010H24Rik	40	Sbsn

**Table A2:** Top 40 most important genes for the EB classifier using a neocortex data analysis.

Ranking	Gene	Ranking	Gene
1	Gm49327	21	Uevld
2	Cirbp	22	Hexim2
3	Txnip	23	Itih2
4	Igfbp3	24	Tsga10
5	Pla2g3	25	Rnpep
6	Cbln3	26	Alpl
7	Fkbp5	27	Steap3
8	Gm20521	28	Lyg2
9	Col23a1	29	Vstm5
10	Gm30003	30	H3f3aos
11	Gm5914	31	Gm10570
12	Slc25a34	32	Ces2b
13	Cdkl3	33	Gm45548
14	Fcna	34	Gm45453
15	F830212C03Rik	35	Rsph10b
16	Scgb3a1	36	Gm7628
17	Hif3a	37	Wrn
18	Asprv1	38	Gm14252
19	Slco2a1	39	Lsp1
20	Klhdc9	40	Slc6a12

The ranking of the top 40 genes using the EB method on the neocortex data; EB: empirical Bayes estimate; Neocortex: neocortex RNA-seq data.

**Table A3:** Top 40 most important genes for the EB classifier using the weighted gene method.

Ranking	Gene	Weight	Ranking	Gene	Weight
1	Gm13230	0.94847	21	Ighg3	0.98984
2	Zfp990	0.96008	22	Paqr5	0.51213
3	Tmprss11d	0.98508	23	Txnip	0.06262
4	Eno1b	0.92767	24	1810010H24Rik	0.92349
5	Hsph1	0.50443	25	Ephx2	0.50384
6	Alpl	0.50826	26	Mt2	0.94691
7	Masp2	0.50653	27	Myoc	0.92971
8	Gm29367	0.92111	28	Rex2	0.95681
9	Cirbp	0.49959	29	Amy1	0.51611
10	Slc25a34	0.50247	30	Map3k6	0.50406
11	Slco2a1	0.50414	31	Krt28	0.52644
12	Miip	0.93724	32	Igsf1	0.98553
13	Igfbp3	0.48639	33	Gm17244	0.51114
14	Elf5	0.93675	34	Kirrel2	0.99110
15	Tnfrsf8	0.94475	35	Ces2b	0.50348
16	Sult1a1	0.93020	36	Acer2	0.93197
17	Tekt4	0.92519	37	Fkbp5	0.50177
18	Zfp992	0.94891	38	Gm9925	0.50643
19	Cnmd	0.97520	39	Pxmp2	0.92845
20	Gm43150	0.48275	40	Gm5914	0.49911

**Table A4:** Top 40 most important genes for the EB classifier using the weighted *Z* method.

Ranking	Gene	Weight	Ranking	Gene	Weight
1	Cirbp	0.49072	21	Igfbp3	0.46329
2	Slc25a34	0.54211	22	Acer2	0.56409
3	Miip	0.61340	23	Paqr5	0.55760
4	Fkbp5	0.51282	24	Ak4	0.51710
5	Gm29367	0.61020	25	Gm13071	0.54671
6	Sult1a1	0.60701	26	Elf5	0.67904
7	Gm13230	0.69359	27	Zfp992	0.71311
8	Zfp990	0.75402	28	Sbsn	0.56089
9	Eno1b	0.66139	29	Cd180	0.52914
10	Alpl	0.59642	30	Ighg3	0.91566
11	Gm17244	0.58839	31	Gkn3	0.51397
12	Masp2	0.55767	32	Gm7628	0.52943
13	Hsph1	0.59928	33	Txnip	0.40891
14	Tekt4	0.60080	34	Rsph10b	0.52757
15	Tnfrsf8	0.64046	35	Znf41-ps	0.54300
16	Map3k6	0.56794	36	Gm9925	0.53913
17	Gm5914	0.50121	37	Cnmd	0.77883
18	1810010H24Rik	0.57946	38	Kirrel2	0.92445
19	Tmprss11d	0.88090	39	Apln	0.49753
20	Ces2b	0.50177	40	Hif3a	0.50933

The ranking of the top 40 genes using the EB method on the weighted *Z* scores: the weighted *Z* scores using the score of the spinal cord and neocortex data; EB: empirical Bayes estimate.

**Table A5:** The distribution of mice in terms of gender.

Gender	Normal Group	Disease Group
Male (n)	4	4
Female (n)	4	4
